# Estimating attrition in mild-to-moderate Alzheimer’s disease and mild cognitive impairment clinical trials

**DOI:** 10.1186/s13195-023-01352-0

**Published:** 2023-11-21

**Authors:** Marina Ritchie, Daniel L. Gillen, Joshua D. Grill

**Affiliations:** 1 UC Irvine Institute for Memory Impairments and Neurological Disorders, University of California, Irvine, Irvine, CA 92697 USA; 2 Department of Neurobiology and Behavior, University of California, Irvine, Irvine, CA 92697 USA; 3 Department of Statistics, University of California, Irvine, Irvine, CA 92697 USA; 4 Department of Psychiatry and Human Behavior, University of California, Irvine, Irvine, CA 92697 USA

**Keywords:** Retention, Alzheimer’s disease, Clinical trials, Trial duration

## Abstract

**Background:**

Participant retention is a key factor that affects clinical trial integrity. Trial protocols estimate attrition as a function of sample size calculations. Alzheimer’s disease (AD) is an area of active treatment development. We aimed to quantify the association between trial duration and completion rates and provide guidance for estimating attrition in AD trial protocols.

**Methods:**

Using the Alzforum and ClinicalTrials.gov databases, we analyzed retention data from 125 mild-to-moderate AD and 12 mild cognitive impairment (MCI) clinical trials. We compared the rates of completion between trial arms (active vs. control) and ran regression models to test the hypothesis that trials with longer study duration have lower trial completion using all available data and restricting to placebo data. Our primary outcome was the odds of trial completion for a 6-month increase in trial duration. From the regression model, we estimated the proportion of participants completing 6-, 12-, and 18-month trials.

**Results:**

We found that 21 (17%) mild-to-moderate AD trials and 1 (8%) MCI trial demonstrated greater dropout in treatment compared to placebo arms. For every 6-month increase in trial duration, there was a 27% decrease in the odds of trial completion (OR = 0.73; 95% CI 0.66, 0.81; *p* < 0.001) among participants in mild-to-moderate AD trials and a 55% decrease (OR = 0.45; 95% CI 0.36, 0.57; *p* < 0.001) among participants in MCI trials. The proportion of participants in the placebo group completing 6-, 12-, and 18-month trials were estimated to be 85.2%, 80.0%, and 73.3% for mild-to-moderate AD trials and 91.9%, 84.2%, and 71.3% for MCI trials, respectively.

**Conclusions:**

Longer duration trials may be underpowered to demonstrate estimated treatment effects and may suffer from a greater risk of bias than do shorter trials.

**Supplementary Information:**

The online version contains supplementary material available at 10.1186/s13195-023-01352-0.

## Introduction

Participant retention directly affects the validity and generalizability of clinical trial results. Greater than anticipated dropout can lead to failed futility analyses, extended or prematurely terminated trials, and invalid or biased results [1–3]. Trials with greater than anticipated dropout are also at risk of being underpowered and unethical [4]. Guidelines from the American Academy of Neurology classify trial results with lower than 80% participant retention to be lower-quality evidence [5]. Accounting for anticipated dropout is critical to ensuring sufficient statistical power to detect an intervention effect if one exists [4, 6]. Research investigating how trial design features impact retention, however, remains limited. For example, limited information is available for expected attrition for trials of varying durations. There is a need for evidence-based retention estimates to guide trialists. To provide such estimates, we examined the impact of trial duration on study completion in mild-to-moderate Alzheimer’s disease (AD) and mild cognitive impairment (MCI) trials. AD is an active area of drug development and trials in this area face unique challenges in participant retention [7].

## Methods

Using the AlzForum and ClinicalTrials.gov databases, we reviewed phase II and phase III placebo-controlled trials conducted between 1995 and 2022 (Table 1). We included parallel design trials with results available on ClinicalTrials.gov or in a peer-reviewed publication and excluded trials that terminated before completion. Trials with primary outcome measures that could add heterogeneity to our estimates (i.e., depression, sleep disturbance, agitation, psychosis) were excluded. We also excluded randomized withdrawal trials, crossover design studies, and trials with a primary outcome of time to conversion. When trials had a range of possible follow-up durations, we used the average reported duration. Trials with two cohorts of different trial durations were included as separate data points. We assessed only the double-blind phase of a study. We categorized trials into MCI or mild-to-moderate AD. The MCI analyses included studies that enrolled participants with MCI (based exclusively on clinical and cognitive diagnostic criteria) or “prodromal AD” (diagnostic criteria for MCI plus a biomarker for AD). Mild-to-moderate AD analyses included trials enrolling participants with “early AD” (frequently including mild dementia and MCI with a biomarker for AD), “mild AD dementia,” and “mild-to-moderate AD dementia” (typically based on diagnostic criteria and scores on the Mini-Mental State Exam).

**Table 1:** Criteria for inclusion

Inclusion criteria
Phase II or phase III
Placebo-controlled
Conducted between 1995 and 2022
Results available on ClinicalTrials.gov or in a peer-reviewed publication
Include patients meeting MCI or mild-to-moderate AD criteria (early AD, mild AD dementia, and mild-to-moderate AD dementia trials)
Exclusion criteria
Terminated before completion
Primary outcome measures that could add heterogeneity to estimates (i.e., depression, sleep disturbance, agitation, psychosis, apathy)
Primary outcome measure of time to conversion
Randomized withdrawal trials
Crossover design trials
Trials enrolling participants with different types of dementia (i.e., mixed dementia, vascular dementia)
Open-label trials
Primary objective to assess the uptake, safety, reliability, or accuracy of positron emission tomography (PET) scans (or PET tracers)

### Statistical analyses

For trials that reported results by trial arm, we assessed the frequency with which trial arms differed in overall retention. When trials included more than one active arm, we combined those arms into a single group. To model associations between trial duration and retention, we used binomial regression. Robust variance estimates were used for all inferences to account for potential deviations from the binomial model mean–variance relationship. We ran the model using data from all participants (in the treatment and placebo groups) and with only the placebo group to remove potential bias due to treatment effects. Trials with no breakdown of participant trial completion by treatment group were removed from the placebo analysis. Based on the regression model, we estimated the proportion of participants completing 6-, 12-, and 18-month trials for MCI and mild-to-moderate AD trials. As exploratory analyses, we ran subgroup analyses by trial characteristics including, trial phase (phase II vs. III), therapeutic purpose (disease-modifying vs. symptomatic treatment), and trial site (single- vs. multi-site).

## Results

**Fig. 1:**
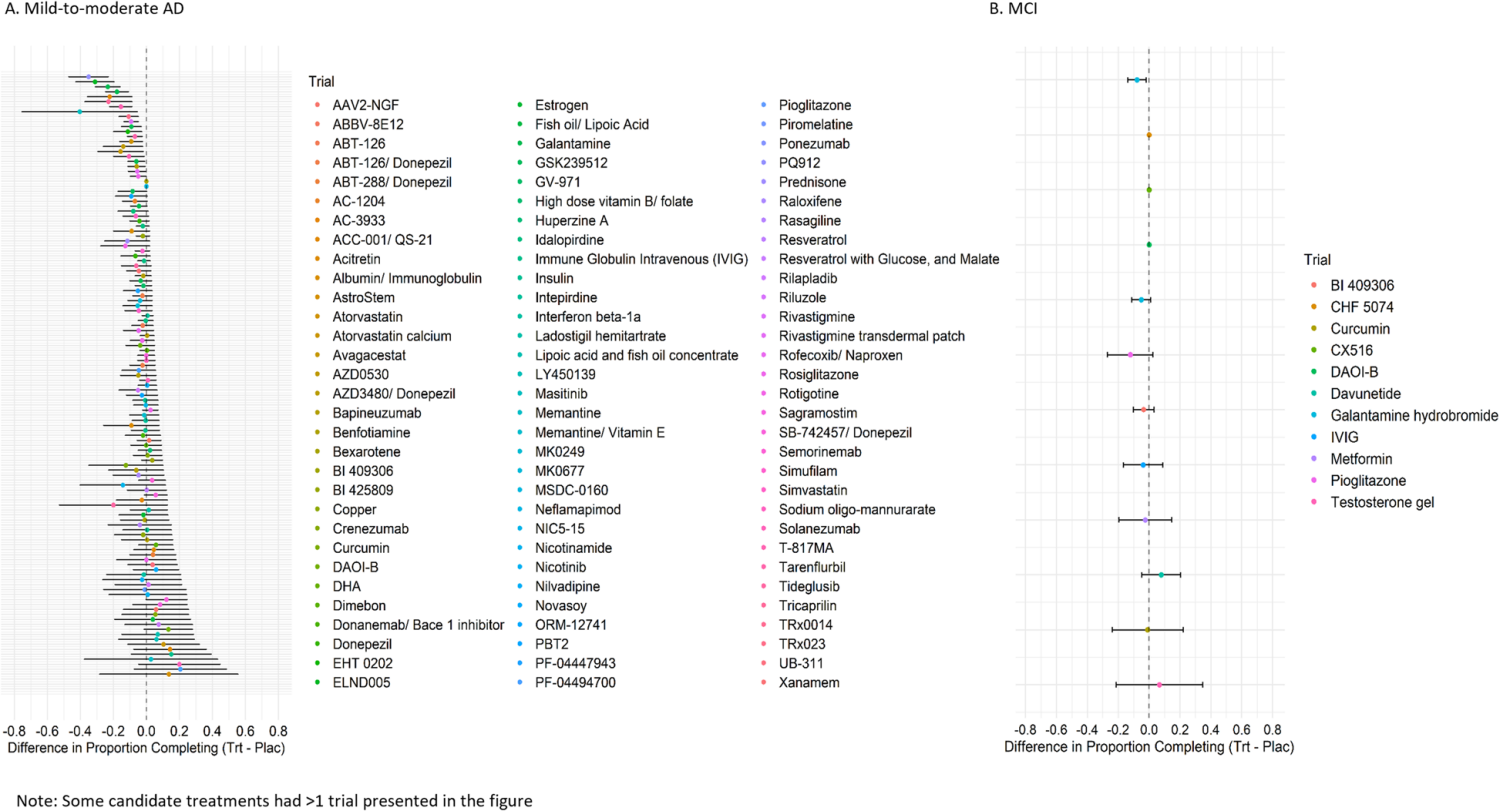
Treatment and placebo group differences in the observed proportion of trial completion

One hundred twenty-five mild-to-moderate AD and twelve MCI trials met the criteria for inclusion in this study. Three mild-to-moderate AD trials did not report differences in completion by treatment arms. We found that 21 mild-to-moderate AD trials and 1 MCI trial demonstrated greater dropout in the treatment arm (Fig. 1). No trials were observed to have greater dropout in the placebo arm.

**Fig. 2:**
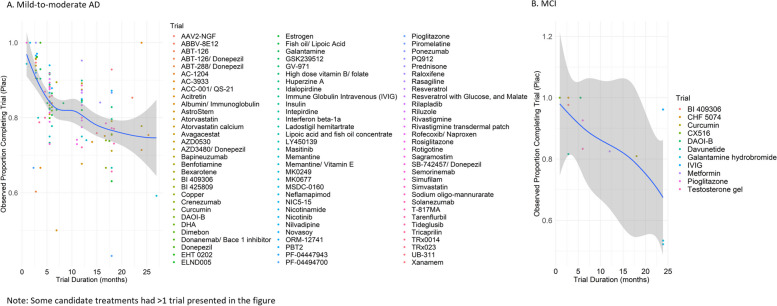
Observed proportion of trial completion by trial duration

Trials with longer study duration had lower retention (Fig. 2). Using binomial regression with robust variance estimates to account for within-trial correlation, we estimated that a 6-month increase in trial duration was associated with a 27% decrease in the odds of trial completion (OR = 0.73; 95% CI 0.66, 0.81; *p* < 0.001) among mild-to-moderate AD trials and a 55% decrease (OR = 0.45; 95% CI 0.36, 0.57; *p* < 0.001) among MCI trials. In regression models including participants in both treatment and placebo groups, completion rates for 6-, 12-, and 18-month trials were estimated to be 82.6%, 77.5%, and 71.4% for mild-to-moderate AD trials and 91.7%, 83.3%, and 69.3% for MCI trials, respectively. In models including only participants in placebo groups, the completion rates for 6-, 12-, and 18-month trials were estimated to be 85.2%, 80.0%, and 73.3% for mild-to-moderate AD trials and 91.9%, 84.2%, and 71.3% for MCI trials, respectively. In exploratory analyses, we observed fairly consistent associations between trial duration and completion rates for subgroups of mild-to-moderate AD trials (sample size prevented subgroup analyses in MCI trials). Rates were similar for phase II (*n* = 84; OR = 0.72; 95% CI 0.59, 0.88; *p* = 0.002) and phase III (*n* = 39; OR = 0.74; 95% CI 0.66, 0.83; *p* < 0.001) trials; for disease-modifying (*n* = 89; OR = 0.77; 95% CI 0.69, 0.87; *p* < 0.001) and symptomatic (*n* = 35; OR = 0.42; 95% CI 0.15, 1.22; *p* = 0.1094) trials; and for single-site (*n* = 17; OR = 0.81; 95% CI 0.60, 1.08; *p* = 0.153) and multi-site (*n* = 107; OR = 0.73; 95% CI 0.66, 0.81; *p* < 0.001) trials (Additional file 1: Fig. S1).

## Discussion

In this analysis of MCI and mild-to-moderate AD trials, we found that participant retention was negatively associated with trial duration. The proportions of participants in the placebo group completing 6-, 12-, and 18-month trials were estimated to be 85.2%, 80.0%, and 73.3% for mild-to-moderate AD trials and 91.9%, 84.2%, and 71.3% for MCI trials, respectively. Trials with a duration greater than 6 months that incorporate common attrition estimates of 10% per year or 20% overall, therefore, may be at risk of being underpowered. This is concerning, since AD trials have increased in length over time [8].

While trials in both diagnostic groups presented similar patterns of attrition with increasing duration, mild-to-moderate AD trials were estimated to have lower completion estimates at 6- and 12-months while MCI trials were estimated to have lower completion rates at 18 months. Age may be a risk factor for participant dropout [9, 10], and participants in the mild-to-moderate AD trials are likely to be older than those in MCI trials [11]. AD is also a progressive disease. Previous examinations of data from NIH-funded Alzheimer’s Disease Research Centers (ADRC) identified worsening cognitive impairment as a risk factor for dropout [12]. This may be most relevant in longer MCI trials, where cognitive decline may be accompanied by the onset of functional impairment.

AD trial participants must co-enroll with a study partner [7]. Longer trials bring an added burden for dyads and may require more careful planning and resources to reduce modifiable barriers to trial completion [13]. For example, a previous study observed an association between the number of retention tactics used at NIH-funded ADRCs and rates of retention at 1 and 2 years [14]. Trials that require longer participation may also benefit from adjusting other study design features like reducing/replacing high-burden assessments (e.g., lumbar puncture) and increasing motivating factors such as returning test results [15] and even financial bonuses for trial completion [16].

While our study underscores the association between trial duration and retention, trial duration decisions are complex. For example, financial considerations may influence duration decisions [17]. Trials with objectives related to pharmacodynamics and pharmacokinetics may require shorter durations. Trials that enroll participants at earlier stages of the disease (e.g., MCI or early AD) may observe less change over time, therefore requiring longer durations [18, 19]. Our results suggest relatively similar associations between duration and retention between MCI and dementia trials. Symptomatic agents may produce measurable effects more rapidly, requiring shorter durations, while disease-modifying treatments may require a longer duration to detect measurable attenuation of AD progression [18]. Though the association with duration was similar between symptomatic and disease-modifying trials here, we note that symptomatic trials were heavily skewed toward shorter durations. We also found similar associations between phase II and phase III trials and between single- and multi-site trials. Overall, trial duration should not be determined based on anticipated retention rates; however, our findings suggest that duration does impact retention and can provide valuable insights into projected retention rates for investigators during the design stage.

### Limitations

The retention estimates in this study may not be representative of all MCI and AD trials due to publication bias. We did not adjust the models in our study for other potential confounding factors that may be associated with retention, such as personal characteristics of the participants (e.g., study partner types, race and ethnicity, education) or trial design features (e.g., number of visits, alternate allocation, mode of treatment administration). Trials that were terminated before completion were excluded from our analysis, which could have biased our estimates, particularly if high dropout contributed to early termination. Some of the trials included in our analyses were ongoing during the COVID-19 pandemic, potentially resulting in a change in their protocols, including adopting a decentralized approach or extending trial duration. We were unable to assess how such changes affected retention estimates.

## Conclusions

While decisions related to expected trial attrition are guided by several factors, these estimates may assist investigators in designing trials and suggest that attrition is associated with trial duration and may frequently exceed protocolized expectations.

### Supplementary Information

**Additional file 1: Fig. S1.** The effect of trial duration on the observed proportion of participants completing a trial stratified by a) trial phase (phase II vs. phase III), b) therapeutic purpose (disease-modifying vs. symptomatic treatments), and c) site (multi- vs. single-site).

## Data Availability

The datasets analyzed during this study are available on ClinicalTrials.gov or in peer-reviewed publications and its supplementary information files.

## Abbreviations

AD: Alzheimer’s disease
ADRC: Alzheimer’s Disease Research Centers
CI: Confidence interval
MCI: Mild cognitive impairment
OR: Odds ratio
PET: Positron emission tomography
NIH: National Institutes of Health
