# Supplementary figures and images for: Estimating attrition in mild-to-moderate Alzheimer’s disease and mild cognitive impairment clinical trials
Source: Alzheimers Res Ther. 2023 Nov 21;15:203. doi: 10.1186/s13195-023-01352-0 (PMC10662394; doi:10.1186/s13195-023-01352-0)

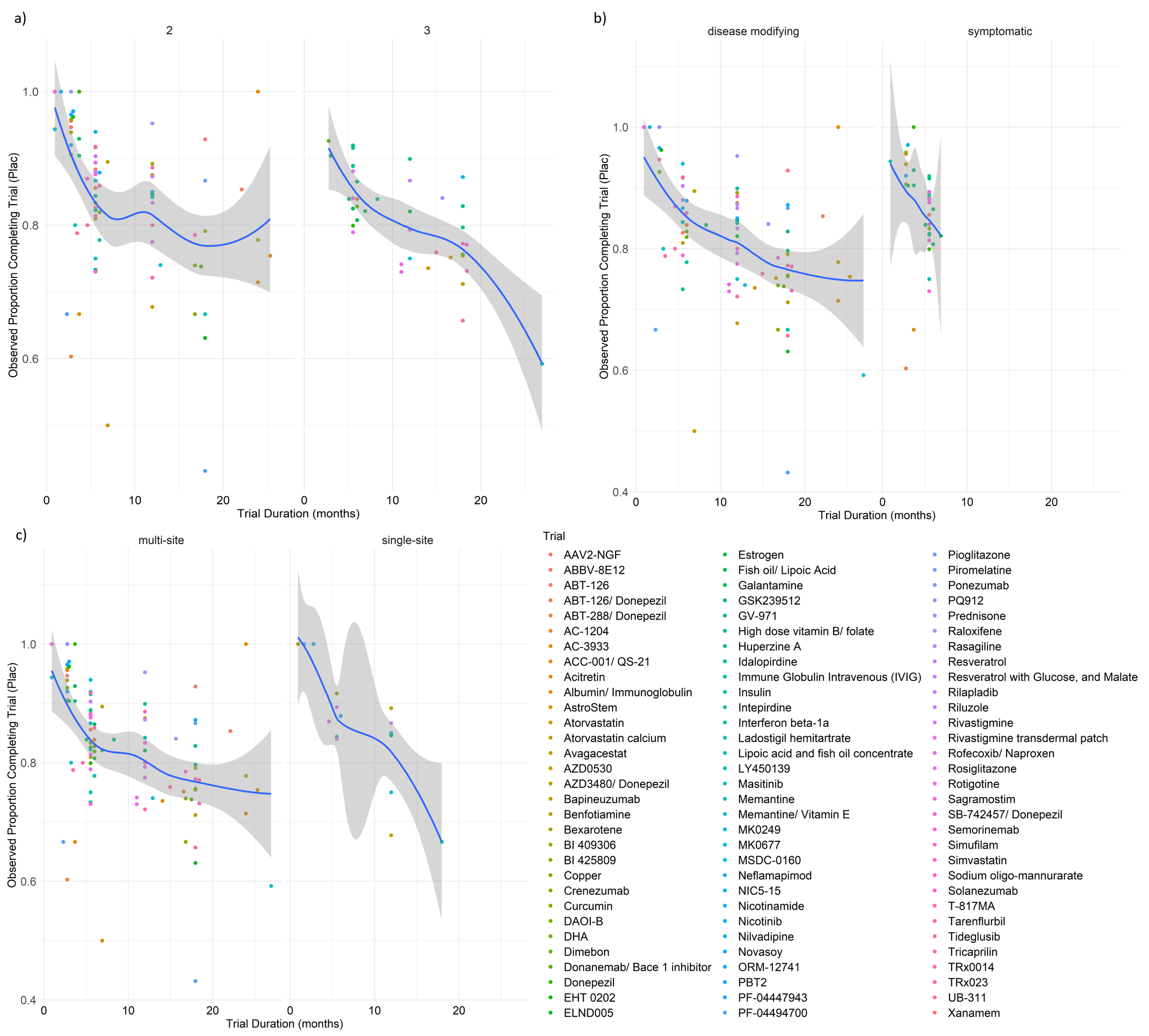

Supplement: Supplementary file 1 — Additional file 1: Fig. S1. The effect of trial duration on the observed proportion of participants completing a trial stratified by a) trial phase (phase II vs. phase III), b) therapeutic purpose (disease-modifying vs. symptomatic treatments), and c) site (multi- vs. single-site). [file 13195_2023_1352_MOESM1_ESM.png]
